# Effects of Nd:YAG laser irradiation on the growth of *Candida albicans* and *Streptococcus mutans*: in vitro study

**DOI:** 10.1007/s10103-018-2622-6

**Published:** 2018-08-25

**Authors:** Kinga Grzech-Leśniak, Joanna Nowicka, Magdalena Pajączkowska, Jacek Matys, Maria Szymonowicz, Piotr Kuropka, Zbigniew Rybak, Maciej Dobrzyński, Marzena Dominiak

**Affiliations:** 1 Department Oral Surgery, Wroclaw Medical University, Krakowska 26, 50-425 Wrocław, Poland; 2 Department of Microbiology, Faculty of Medicine, Wroclaw Medical University, Chałubińskiego 4, 50-368 Wrocław, Poland; 3 Private Dental Practice, Lipowa 18, 67-400 Wschowa, Poland; 4 “Sapienza” University of Rome, Rome, Italy; 5 Department of Experimental Surgery and Biomaterials Research, Wroclaw Medical University, Bujwida 44, 50-345 Wrocław, Poland; 6 Department of Histology and Embriology, Wroclaw University of Environmental and Life Sciences, Norwida 31, 50-375 Wrocław, Poland; 7 Department of Conservative Dentistry and Pedodontics, Wroclaw Medical University, Krakowska 26, 50-425 Wrocław, Poland

**Keywords:** Biofilm, Cell metabolism, Flat-top handpiece, LLLT, Neodymium laser

## Abstract

The purpose of this study was to evaluate the effects of Nd:YAG laser with flat-top handpiece on the in vitro growth of *Candida albicans* and *Streptococcus mutans*. The incidence of *C. albicans* (opportunistic commensal) and *S. mutans* (facultatively anaerobic) infections is increasing, despite available treatments. Cultures of *Streptococcus mutans* and *Candida albicans* were irradiated using Nd:YAG laser (LightWalker, Fotona) with flat-top handpiece (Genova, LightWalker, Fotona) at the following parameters: group G1: 0.25 W, 10 Hz, 15 s, 3 J and group G2: 1 W, 10 Hz, 60s, 59 J. The results were evaluated directly and 24 h after irradiation using a quantitative culture method (estimation of colony-forming units in 1 ml of suspension, cfu/ml), and microscopic analysis with Janus green stain and compared with control group in which laser was not applied. *C. albicans* was reduced by 20 up to 54% for G1, and for G2 by 10 up to 60% directly after the application. The cfu/ml values for *S. mutans* decreased by 13% (*p* = 0.1771) for G1 and 89% (*p* < 0.0001) for G2. In both test groups 24 h after the application, the number of colony-forming units decreased by 15–46% for G1 and by 15–64% for G2. The arrested cell division, increasing the surface area and increasing the number of metabolically inactive cells, were observed in morphometric analysis. Macroscopic and microscopic analyses revealed a reduction in cell number and a significant decrease of cell metabolism after laser application for both *C. albicans* and *S. mutans*.

## Introduction

The human oral cavity is a conducive environment to unrestricted formation of natural microbial biofilm. However, in a distorted equilibrium balance of the oral health, infectious pathogens may gain access into the dental tissues and gingival area. Infectious pathogens from the oral cavity cause oral diseases such as caries, gingivitis, periodontitis, endodontic infections, and alveolar osteitis, and sometimes are concomitant to systemic diseases, including cardiovascular disorders, stroke, preterm birth, diabetes, and pneumonia, among others [1, 2]. *Streptococcus mutans* is one of the main bacterial strains colonizing the oral cavity and a major contributor of tooth decay, which in turn may affect the overall health of the host [3]. The fungal species of *Candida albicans* is by far the most commonly detected fungal organism in humans, part of the healthy human microbiota, but in immunocompromised hosts, it may cause a number of infections, ranging from superficial infections of the mucosa and skin to life-threatening systemic infections. *C. albicans* cells are frequently found along with *S. mutans*-derived plaque biofilms*.* Recent studies indicate high prevalence of *S. mutans* in dental biofilm where the fungal pathogen *C. albicans* resides, suggesting that this association is involved in the enhancement of biofilm virulence [4, 5]. *C. albicans* coadheres with *S. mutans* in the presence of sucrose [5–7]. Such bacterium-fungus association may enhance *S. mutans* infection [8] and augment fungal carriage and infectivity of mucosal disease [9].

Management of infections caused by bacteria and fungi is a viable challenge in various medical fields, including dentistry. It cannot be emphasized enough that we need to focus on the search for alternative methods to manage rapidly developing drug resistance and recurrent candidiasis [10]. The development of laser medicine has provided a number of new therapy modalities capable of damaging pathogenic organisms. Photoantimicrobial therapy is safe, effective, and easy to implement and its activity spectrum covers bacteria, fungi, viruses, and protozoa, which make it superior to conventional therapies [11]. There are studies reporting antimicrobial and bactericidal effects of laser application [10]. Among them, one promising treatment modality is low-level laser therapy (LLLT), in which non-thermal laser irradiation is applied to the target site. LLLT uses mainly semiconductor lasers at 685 and 830 nm wavelengths, which are considered safe and effective against biofilm-associated infections [10]. In this study, Nd:YAG laser at 1064 nm was used based on the assumption that its characteristic qualities, high scattering effect, and deep soft tissue penetration could be more effective in reduction of *C. albicans* and *S. mutans*.

Antibacterial effects of Nd:YAG laser in patients treated for oral diseases have been confirmed in previous research, where pulsed Nd:YAG laser was applied with power settings over 2 W to induce a photothermal effect [12–14]. The efficacy of laser irradiation has been demonstrated against *Escherichia coli*, *Staphylococcus aureus*, *Actinomyces naeslundii*, *Pseudomonas aeruginosa*, *Enterococcus faecalis*, and *Streptococcus anginosus* [12, 15, 16].

The present study aimed at evaluating the effects of laser therapy on pathogenic organisms commonly inhabiting the oral cavity using Nd:YAG with a flat-top handpiece. Flat-top handpiece spreads the energy of the laser beam evenly over the treated area in a no contact mode. It can be used at a variable distance of up to 100 cm without modifying the irradiation energy over a 1-cm spot diameter. The handpiece is most commonly used for biostimulation and anti-inflammatory treatment. The present study explored the potential of Nd:YAG laser with flat-top handpiece to affect the in vitro growth of *S. mutans* and *C. albicans*—the two oral microbials which may become pathogenic in the presence of predisposing factors, producing infections that range from local to systemic.

## Material and methods

To evaluate the effect of laser on selected cultures, quantitative culture technique (indicating the number of colony-forming units [cfu]/ml) and microscopic analysis with Janus green stain were used. All tested and control samples were subjected to triplicate procedure.

### Samples preparation

Reference cultures of *S. mutans* (ATCC 25175) and *C. albicans* (ATCC 90028) and 3 clinical cultures of *C. albicans* collected from throat swab were selected for analysis (ethical approval by Research Ethics Board no. KB 342/2018). *S. mutans* and *C. albicans* colonies were subcultured from vial stock and a suspension was prepared (0.5 McFarland standard). *S. mutans* samples were inoculated in Columbia Agar with 5% sheep blood and incubated for 48 h at 37 °C and increased level of CO2. *C. albicans* samples were inoculated in Sabouraud’s dextrose agar for 48 h at 37 °C. Preparation of control inocula of *S. mutans* and *C. albicans*, respectively, followed the same procedure.

### Laser application

The present study used a near-infrared neodymium-doped yttrium aluminum garnet, Nd:YAG laser (LightWalker, Fotona, Slovenia) at 1064 nm wavelength with flat-top handpiece (Genova, LightWalker, Fotona, Slovenia). The handpiece produces a spot size with a homogeneous beam profile of Nd:YAG laser light.

The investigation included two sets of parameters, changing the power and duration of irradiation. The following parameters were applied in two experimental groups:

Group 1 (G1): Genova handpiece, operation mode: pulsed wave (PW), power 0.25 W in non-contact mode, frequency10 Hz, energy 25 mJ, distance 10 mm, fluence per pulse 0.03 J/cm^2^, power density per second 0.32 W/cm^2^, spot diameter 10 mm, time15 s, total dose 3 J (measured by Nd:YAG laser software).

Group 2 (G2): Genova handpiece, operation mode: pulsed wave (PW), power 1 W in non-contact mode, frequency10 Hz, energy 100 mJ, distance 10 mm, fluence per pulse 0.13 J/cm^2^, power density per second 1.27 W/cm^2^, spot diameter 10 mm, time 60 s, total dose 59 J (measured by Nd:YAG laser software) (Table 1).

**Table 1:** Nd:YAG laser parameters used in the study

Study group	Handpiece	Distance (mm)	Energy (mJ)	Power (W)	Frequency (Hz)	Spot (mm)	Fluence (J/cm^2^)	Power density (W/cm^2^)	Total dose (J)
G1	Genova	10	25	0.25	10	10	0.03	0.32	3
G2	Genova	10	100	1	10	10	0.13	1.27	59

*cm*^*2*^ square centimeter, *Hz* Hertz, *J* Joule, *mJ* millijoule, *mm* millimeter; *W* watt

Control group: non-irradiated reference and clinical cultures of *S.mutans* and *C.albicans*.

### Microorganism quantification

The effect of laser on microorganisms (their viability in particular) was evaluated directly after laser irradiation and at 24 h after the application. One milliliter of each inocula was exposed to laser light at the fixed work distance of 1 cm (distance from light source and cell line surface) and then samples of 100 μl were extracted to be seeded, ranging from 10^−1^ to 10^−8^ cfu/ml. After incubation time macroscopic evaluation of the samples was performed to determine the cfu/ml value. Preparation of control inocula of *S. mutans* and *C. albicans*, respectively, followed the same procedure as described for experimental samples, but they were not irradiated.

To evaluate the effect of laser light on the number of cells the cfu/ml value for control samples was assumed 100%. The change of cell number was calculated on the basis of:

$$ Z\mathrm{cfu}\%=100\%-\left(\frac{\mathrm{cfuL}}{\mathrm{cfuK}}\ast 100\%\right) $$

*Z*cfu%: % change of microorganism cells number (cfu/ml) after laser application
cfuL: Value of colony-forming units in test samples (G1, G2) after laser application
cfuK: Value of colony-forming units in control group

### Microscopic analysis with Janus green stain

To ensure unbiased results, the researcher responsible for microscopic analysis was not informed of the content of the samples (double-blind trial). From the fresh culture of the analyzed strains, microorganism suspension concentration at 0.5 in McFarland Standard was prepared. One milliliter of such suspension was treated with laser (G1 and G2), centrifuged, and the sediment was applied on microscopic slide. After incubation time of 5 min in 1% of Janus green, the material was washed under tap water and covered by glass. Then, material was analyzed under microscope (Eclipse 80i, Nikon Instruments Inc., USA) in normal and polarized light. Morphometric analysis was performed with the use of Nis-Elements AR (Nikon Instruments Inc., USA) software.

The microscopic analysis of the laser impact on microorganisms was performed directly after laser irradiation and after 24 h. The control group was a suspension of the analyzed microorganisms prepared as above but not irradiated with laser light.

### Statistical analysis

Data were analyzed using Analysis Tool Pack for MS Excel (Microsoft Corporation, Redmond, Washington, United States) with *α* = 0.05. Mann-Whitney test was used to analyze differences between test and control groups, between test groups and between reference and clinical colonies directly after laser application and 24 h after. To evaluate the significance of change between the given time points, Wilcoxon test was used to compare paired data. To ensure unbiased results, the researcher responsible for statistical analysis was not informed which data derived from macroscopic analysis referred to test and control groups (double-blind trial).

## Results

### Quantitative analysis of the effect of laser on tested samples

Directly after laser application on reference culture of *S. mutans*, the recorded values of cfu/ml were 3.5 × 10^8^ and 9.40 × 10^8^ for G1 and G2, respectively. Twenty-four hours after the application, the values were 2.6 × 10^8^ (G1) and 4.02 × 10^8^ (G2) (Table 2).

**Table 2:** The effect of laser on *S. mutans* viability (reference culture)

*Streptococcus mutans* (ATCC 25175)			
G1 (0.25 W, 10 Hz, 15 s, 3 J/cm^2^)		G2 (1 W, 10 Hz, s, 59 J/cm^2^)	
Control	4 × 10^8^ cfu/ml	Control	8.90 × 10^9^ cfu/ml
Directly after irradiation	3.5 × 10^8^ cfu/ml	Directly after irradiation	9.40 × 10^8^ cfu/ml
Control after 24 h	4.5 × 10^8^ cfu/ml	Control after 24 h	7.40 × 10^9^ cfu/ml
At 24 h after irradiation	2.6 × 10^8^ cfu/ml	At 24 h after irradiation	4.02 × 10^9^ cfu/ml

Directly after laser application on reference and clinical cultures of *C. albicans*, the recorded values of cfu/ml were in the range of 8 × 10^5^–4.6 × 10^6^ for G1 and 2 × 10^5^–5.6 × 10^6^ for G2. Evaluating cell viability 24 h after the application, the following values were recorded: 1.44 × 10^8^–7.95 × 10^8^cfu and 7.4 × 10^7^–7.2 × 10^9^ for G1 and G2, respectively (Tables 3, 4, 5, and 6).

**Table 3:** The effect of laser on *C. albicans* viability (reference culture)

*Candida albicans* (ATCC 90028)			
G1 (0.25 W, 10 Hz, 15 s, 3 J/cm^2^)		G2 (1 W, 10 Hz, 60s, 59 J/cm^2^)	
Control	3.84 × 10^6^ cfu/ml	Control	3.32 × 10^6^ cfu/ml
Directly after irradiation	3.04 × 10^6^ cfu/ml	Directly after irradiation	2.50 × 10^6^ cfu/ml
Control after 24 h	4 × 10^7^ cfu/ml	Control after 24 h	2.73 × 10^8^ cfu/ml
At 24 h after irradiation	3.42 × 10^7^ cfu/ml	At 24 h after irradiation	9.9 × 10^7^ cfu/ml

**Table 4:** The effect of laser on *C. albicans* [1] viability (clinical culture)

*Candida albicans* [1]			
G1 (0.25 W, 10 Hz, 15 s, 3 J/cm^2^)		G2 (1 W, 10 Hz, 60s, 59 J/cm^2^)	
Control	1.01 × 10^7^ cfu/ml	Control	9.3 × 10^6^ cfu/ml
Directly after irradiation	4.6 × 10^6^ cfu/ml	Directly after irradiation	5.6 × 10^6^ cfu/ml
Control after 24 h	3.77 × 10^8^ cfu/ml	Control after 24 h	3.54 × 10^9^ cfu/ml
At 24 h after irradiation	7.95 × 10^8^ cfu/ml	At 24 h after irradiation	7.2 × 10^9^ cfu/ml

**Table 5:** The effect of laser on *C. albicans* [2] viability (clinical culture)

*Candida albicans* [2]			
G1 (0.25 W, 10 Hz, 15 s, 3 J/cm^2^)		G2 (1 W, 10 Hz, 60s, 59 J/cm^2^)	
Control	3.3 × 10^6^ cfu/ml	Control	2 × 10^6^ cfu/ml
Directly after irradiation	3.3 × 10^6^ cfu/ml	Directly after irradiation	1.8 × 10^6^ cfu/ml
Control after 24 h	4.14 × 10^8^ cfu/ml	Control after 24 h	3.93 × 10^8^ cfu/ml
At 24 h after irradiation	2.45 × 10^8^ cfu/ml	At 24 h after irradiation	3.36 × 10^8^ cfu/ml

**Table 6:** The effect of laser on *C. albicans* [3] viability (clinical culture)

*Candida albicans* [3]			
G1 (0.25 W, 10 Hz, 15 s, 3 J/cm^2^)		G2 (1 W, 10 Hz, 60s, 59 J/cm^2^)	
Control	1 × 10^6^ cfu/ml	Control	5 × 10^5^ cfu/ml
Directly after irradiation	8 × 10^5^ cfu/ml	Directly after irradiation	2 × 10^5^ cfu/ml
Control after 24 h	2.67 × 10^8^ cfu/ml	Control after 24 h	1.35 × 10^8^ cfu/ml
At 24 h after irradiation	1.44 × 10^8^ cfu/ml	At 24 h after irradiation	7.4 × 10^7^ cfu/ml

Directly after the application, the cfu/ml values for *S. mutans* decreased by 13% (*p* = 0.1771) for G1 and 89% (*p* < 0.0001) for G2. The viability of *S. mutans* after laser irradiation were reduced by 42% (*p* < 0.0001) for G1 and 46% (*p* < 0.0001) for G2.

**Fig. 1:**
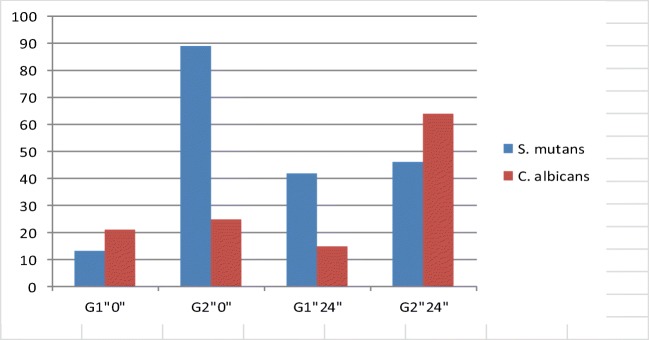
Percent reduction of *S. mutans* and *C. albicans* after the application of laser. G1”0” directly after application; G2”0” directly after application; G1”24” 24 h after application; G2”24” 24 h after application

*C. albicans* cfu/ml values were reduced by 20 up to 54% for G1, and for G2 by 10 up to 60% directly after the application. In both test groups 24 h after the application, the number of colony-forming units decreased by 15–46% for G1 and by 15–64% for G2. Statistically significant reduction of cfu/ml value (*p* < 0.0001) was found for the majority of analyzed *C. albicans* cultures in both test groups (Fig. 1).

### Microscopic analysis

**Fig. 2:**
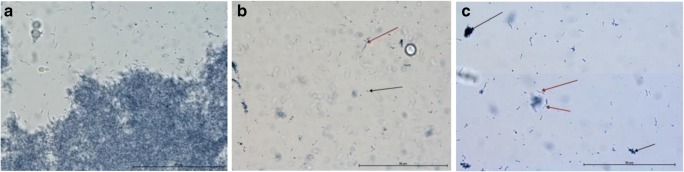
Microscopic analysis of laser effect on reference cultures of *S. mutans* (ATCC 25175). **a** Control, aggregation of metabolically active bacteria; **b** G1, single bacteria (black arrow) and few colony-forming units (red arrow); **c** G2, numerous remnants of dead bacteria (black arrow). Streptococcus chains revealed different level of cell metabolism (red arrow); Janus green Mag. × 1000

Microscopic analysis of *S. mutans* cultures revealed reduction of the cell number and significant reduction in cell activity after laser application for G1. Some of the cells remained intact, but their metabolic activity was lower when compared to control group (Fig. 2b). Similar results were observed after application of laser for G2, where numerous remnants of the death cells remained attached to the slide glass. The metabolic activity of the cells in this group was weaker than in G1 group or there were none (Fig. 2).

**Fig. 3:**
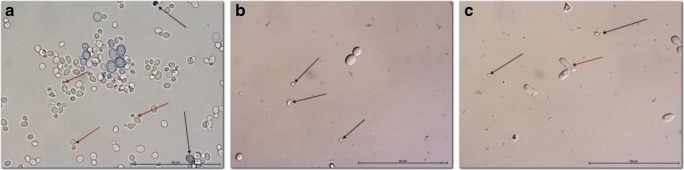
Microscopic analysis of laser effect on clinical cultures of *C. albicans* 2 (sample photos). **a** Control, numerous cells of various sizes. Small cells have a green tinge, which is characteristic of metabolically active cells. They are often accompanied by smaller cells created as a result of budding (red arrow). Larger blue-colored cells (black arrow) display limited metabolic activity or are already inactive and signs of disintegration are visible. Degenerated cells appear in the smear as small, blue objects with unspecified morphology; **b** G1, visible remains of damaged cells (black arrow), single large cells grouped in 2 or 3 do not show the ability to absorb dye; **c** G2, debris from damaged cells. Some cells form clusters of 2–3 oval-shaped cells with separated nucleus (red arrow) and weak metabolic activity; Janus green; magnification of × 1000

As in the case of *S. mutans*, G1 laser application on *C. albicans* strains resulted in the reduction of cells compared to the control. Remnants from dead cells were observed among alive cells. In some cells, the nucleus was clearly visible, in some, it was not. In group G2, the cells started to change shape and numerous elongated cells with centrally situated nucleus were visible. The cells showed a different degree of dye absorption and oxidation (Fig. 3).

**Fig. 4:**
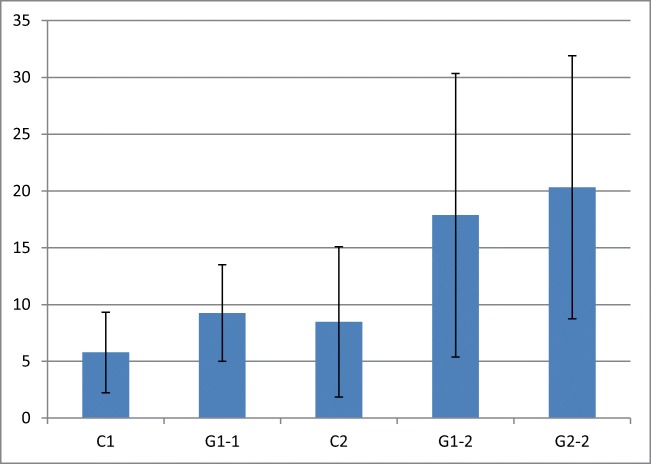
Cell surface (in μm) in the control and test (G1 and G2). Statistical significance calculated for the confidence interval (95%) alpha = 0.05. Clinical strain *C. albicans* 1: C1 control; G1-1 after laser G1; clinical strain *C. albicans* 2: C2 control: G1-2 after laser G1; G2-2 after the G2 laser

**Fig. 5:**
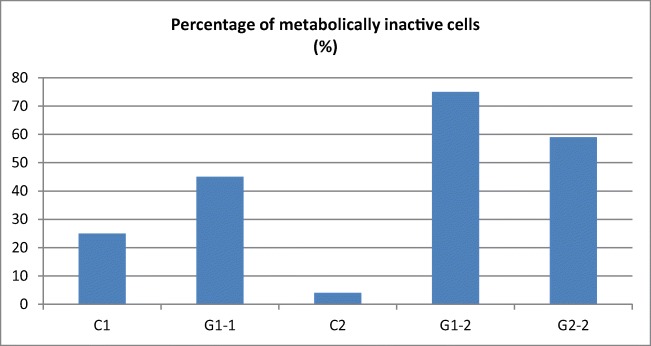
Percentage of metabolically inactive cells in the control and test samples. Clinical strain *C. albicans* 1: C1 control; G1-1 after laser G1; clinical strain *C. albicans* 2: C2 control: G1-2 after laser G1; G2-2 after the G2 laser

Morphometric studies of the cell surface and analysis of metabolically active and inactive cells indicate that the cells are destroyed by the laser light (especially directly after the application). After 24 h, further effects of laser irradiation were observed such as arrested cell division, increasing the surface area and increasing the number of metabolically inactive cells. This is confirmed by morphological observations. The analysis of the effects of laser irradiation revealed a statistically significant reduction (*p* < 0.0001) of both *C. albicans* and *S. mutans* cultures for both sets of parameters of laser application. In both cases (lower power and shorter irradiation (G1) and higher power and longer irradiation (G2)), the number of pathogens was reduced (Figs. 4 and 5, Table 7).

**Table 7:** Statistical significance calculated for the confidence interval (95%) alpha = 0.05

Group/sample	C1	G1-1	C-2	G1-2	G2-2
C1		+	−	+	+
G1-1	+		−	+	+
C2	−	−		+	+
G1-2	+	+	+		−
G2-2	+	+	+	−	

Clinical strain *C. albicans* 1: C1 control; G1-1 after laser G1; Clinical strain *C. albicans* 2: C2 control: G1-2 after laser G1; G2-2 after the G2 laser

Higher power and longer irradiation in G2 provided more reduction of cfu/ml values of 3 cultures of *C. albicans* 24 h after the application than directly after irradiation. The decrease was statistically significant (*p* < 0.05). Such observations were not made for *S. mutans* strains.

Microscopic analysis with Janus green stain revealed changes in the number of analyzed microorganisms and changes in both their metabolic activity and shape. Janus green changes color according to the amount of oxygen present. When oxygen is present, the indicator oxidizes to a blue color. Different levels of dye absorption were observed after the application of G1 and G2 lasers and limited oxidation, which prove reduced cell metabolic activity. This observation was most evident for *Streptococcus* strains, in which inhibited metabolic activity and the change of color to blue were observed. It was also observed that some cells stopped absorbing the dye (lack of color). In the case of fungi, the effect was more profound after the application of lower power and shorter irradiation time (G1). After the application of higher power and longer irradiation time (G2), more cell debris could be seen after their disintegration, which indicates that laser may be more effective for cell destruction rather than inhibition of their activity. It is worth noting that the cells which were still active after the application of laser G2 tend to change shapes, elongate, and prepare to assume different morphological structure.

Laser light affects cell integrity directly after the application and 24 h after further effects could be observed, including inhibited cell division and increased number of metabolically inactive cells. In group G1, reduction of cfu/ml values was observed for all analyzed cultures of *S. mutans* and *C. albicans* directly after the application and 24 h after irradiation. Higher power and longer irradiation time of laser G2 resulted in more reduction of colony-forming units of *S. mutans* and *C. albicans* directly after the application. In group G2, more effective reduction of *C. albicans* strains was observed 24 h after laser irradiation.

## Discussion

Numerous studies on low-level laser therapy in combination with infrared lasers over the last 20 years provided positive results and thus a basis for the introduction of this technology into clinical practice to stimulate wound healing, treat inflammation, and reduce pain [17–19]. However, development of new optical systems and handpieces to photons transport into a beam generated by Nd:YAG laser to the point of application demands verification of the knowledge of using LLLI on cells and tissues. Laser light has also been used in antimicrobial therapy. Studies indicate that laser application can effectively reduce or eliminate pathogenic organisms [20–23]. The study aimed at evaluating the effects of Nd:YAG irradiation on the in vitro growth of *Candida albicans* and *Streptococcus mutans*. The major finding of the study is that Nd:YAG laser with a flat-top handpiece resulted in the reduction of *C. albicans* and *S. mutans* at fluence below the soft tissue ablation threshold (0.03 and 0.13 J/cm^2^). Compared to the control group, irradiation using flat-top handpiece of Nd:YAG laser resulted in the decrease of the cfu/ml value for both *C. albicans* and *S. mutans*. These findings indicate that 1064 nm infrared wavelength is efficient and safe in reduction of the total number of irradiated microorganisms.

Our review of the available literature provides no record of using Nd:YAG laser with the flat-top handpiece in both *C. albicans* and *S. mutans* irradiation. The analysis of the effects of laser irradiation revealed a statistically significant reduction of both *C. albicans* and *S. mutans* cultures for both sets of parameters of laser application. In both cases with a lower dose (3 J at 0.25 W, 10 Hz) or higher dose (59 J at 1 W, 10 Hz), the number of pathogens was reduced.

A recent in vivo study by Piccolo et al. evaluated the effect of Nd:YAG irradiation to fight fungal overgrowth in the nail plate and indicated a great potential for safe and effective clinical application. The use of long-pulsed 1064 nm Nd:YAG laser (neodymium-doped yttrium aluminum garnet) for the treatment of Onychomycosis (caused by *Candida* species, among other pathogenic fungi) has demonstrated promising results [21]. However, the authors used the laser with 5 msec pulse duration and 1 Hz repetition rate at the fluence of 30 J/cm^2^, thus the fungal destruction was evoked strictly by photothermal effects what authors underline in the conclusion.

The use of LLLT with wavelength below 900 nm for *C. albicans* eradication was described in literature [20, 22–24]. Risovic et al. [20] observed the wavelength-dependent eradication efficiency of *C. albicans*, where most effective wavelength for elimination of *C. albicans* was UV-C 254 nm (Δ = 6.1 mJ/cm^−2^, ET99.99 = 56 mJ/cm^−2^) and least efficient 406 nm (Δ = 11.4 J/cm^−2^, ET99.99 = 104 J/cm^−2^). Gupta et al. [24] and Rosa et al. [22] confirmed that violet-blue visible light (400–490 nm) does not require the use of photosensitizer to kill *C. albicans* cells in biofilms. Other studies [10, 23] proved that laser light at 685 nm and 830 nm might be considered a promising treatment for *C. albicans* infections. Also, our present study showed the ability of 1064-nm Nd:YAG laser in eradication *C.albicans* at dose 3.0 J/cm^2^ (0.25 W) and 59 J/cm^2^ (1 W).

Seyedmousavi et al. [10] observed that LLLT with energies > 10 J at both 685 and 830 nm wavelengths produced statistically significant effects in vitro on the pathogenicity of *C. albicans*, and in vivo on the survival rate of mice (*p* value ≤ 0.05). In turn, Maver-Biscanin et al. [23] in their in vivo study reduction of *C. albicans* species in the treated areas were irradiated with different exposure times—5 min (830 nm, 3.0 J/cm^2^, 60 mW) and 10 min (685 nm, 3.0 J/cm^2^, 30 mW). In contrast to above-mentioned studies [10, 23] efficient eradication of *C. albicans* and *S. mutans* was found with an exposure time 15 s (3.0 J/cm^2^, 250 mW) and 60 s (59 J/cm^2^, 1000 mW).

In the literature was described very well that the laser light generated by the infrared lasers with wavelengths in the range of 600–1100 nm affects a wider cell-light response [25]. The dose-dependent effects of LLLT are described by the Arndt-Schultz’s curve [25, 26]. It suggests that different types of stimuli evoke different reactions of cells, e.g., increased stimulus inhibits activity [26]. On the other hand, the bacterial eradication can be obtained in a different process by photodynamic therapy where the irradiated photosensitizer generates induction of reactive oxygen species (ROS) which have a high killing potential for bacteria, fungus, and viruses. Despite the above-mentioned photodynamic effects of LLLT, literature review provides numerous studies on the influence of light on stimulation and inhibition of bacterial growth in the dose range of 1–10 J/cm^2^ without administration of photosensitizers [10]. This effect was defined by the generation of ROS elicited by activation of endogenous chromophores in cells by the infrared laser light [10]. Thus, the results of our study where the irradiation with the two different doses (3 J at 0.25 W/10 Hz or 59 J at 1 W/10 Hz) resulted in a reduction in S. mutans and *C. albicans* amount in the same session can be explained by this above-mentioned phenomenon. The effect of laser on bacterial/fungal destruction was also described by Sommer where the expansion and contraction of the intracellular water volume and fluidity generate bidirectional flow [27].

In our opinion, Nd:YAG laser light at 1064 nm which is closed to the third peak wavelength of 1000 nm causes photoexcitation of endogenous microbial porphyrin molecules contained in *S. mutans* and *C. albicans* evoking oxidative damage through reactive oxygen species (ROS).

Translating our data into the clinical setting, we suggest that laser-based antimicrobial treatment can significantly reduce the quantity of *Streptococcus mutans* and *Candida albicans* especially in the treatment of caries and candidiasis.

More clinical studies are needed to evaluate the efficacy of a direct laser-based approach in vivo. Further study of Nd:YAG application with the Genova handpiece to treat bacterial and fungal infection is still needed to assess the efficacy of Nd:YAG 1064 nm laser therapy as a valid treatment for this frequent pathology.

## Conclusions

The present data suggest that laser light at specific wavelengths might have some positive effects on the reduction of *S. mutans* and *C. albicans* infections; however, the clinical studies should be performed. Laser irradiation merits further attention for the many advantages it offers: shorter treatment duration, lower cost of treatment, minimal or no side effects, and an alternative to the systemic administration of antibiotics. Both low- and high-intensity Nd:YAG laser can significantly reduce the quantity of *Streptococcus mutans* and *Candida albicans*, which can be obtained after further clinical studies.
